# Muscle function in glenohumeral joint stability during lifting task

**DOI:** 10.1371/journal.pone.0189406

**Published:** 2017-12-15

**Authors:** Yoann Blache, Mickaël Begon, Benjamin Michaud, Landry Desmoulins, Paul Allard, Fabien Dal Maso

**Affiliations:** 1 Laboratoire Interuniversitaire de Biologie de la Motricité, Université Lyon 1, Université de Lyon, Lyon, France; 2 Laboratoire de Simulation et Modélisation du Mouvement, Département de Kinésiologie, Université de Montréal, Québec, Canada; Rijksuniversiteit Groningen, NETHERLANDS

## Abstract

Ensuring glenohumeral stability during repetitive lifting tasks is a key factor to reduce the risk of shoulder injuries. Nevertheless, the literature reveals some lack concerning the assessment of the muscles that ensure glenohumeral stability during specific lifting tasks. Therefore, the purpose of this study was to assess the stabilization function of shoulder muscles during a lifting task. Kinematics and muscle electromyograms (n = 9) were recorded from 13 healthy adults during a bi-manual lifting task performed from the hip to the shoulder level. A generic upper-limb OpenSim model was implemented to simulate glenohumeral stability and instability by performing static optimizations with and without glenohumeral stability constraints. This procedure enabled to compute the level of shoulder muscle activity and forces in the two conditions. Without the stability constraint, the simulated movement was unstable during 74%±16% of the time. The force of the supraspinatus was significantly increased of 107% (p<0.002) when the glenohumeral stability constraint was implemented. The increased supraspinatus force led to greater compressive force (p<0.001) and smaller shear force (p<0.001), which contributed to improved glenohumeral stability. It was concluded that the supraspinatus may be the main contributor to glenohumeral stability during lifting task.

## Introduction

The glenohumeral joint enables the greatest articular mobility in the human body at the expense of its stability [1–3]. Clinical instability is usually defined as any translation of the humeral head [4, 5] while, from a biomechanical point of view, glenohumeral joint instability occurs when the resultant glenohumeral reaction force points outside the glenoid surface [6].

Although glenohumeral instability may be caused by an external contact, internal forces produced by the muscles surrounding the humeral head may also result in glenohumeral instability. Near the maximal glenohumeral range of motion, translational forces parallel to the glenoid surface are counteracted by passive structures (e.g. capsulo-ligamentar elements, labrum and intra-joint vacuum) [7]. Nevertheless, translational forces may also be observed during the mid-range of humerus movements and therefore cause glenohumeral instability. Especially, Escamilla et al. [8] pointed out that the activation of the anterior deltoid may cause a translation of the humeral head and consequently reduce the stability of the glenohumeral joint. Therefore, movements such as lifting task for which middle and anterior deltoids are the main prime movers [9, 10] may be subject to glenohumeral instability.

It is commonly accepted that the rotator cuff muscles are the main glenohumeral joint stabilizers [5, 11, 12]. However, some patients with severe rotator cuff tears still manage shoulder function with a sufficient stability [13], meaning that glenohumeral stability results from a complex process. While, many studies assessed glenohumeral instability during analytical movements through cadaveric studies [1, 11, 14, 15] or electromyography approaches [16–18], only one study [19] investigated glenohumeral stability during daily living activities such as lifting task. The authors assumed that during a lifting movement, the latissimus dorsi and teres minor stabilize the glenohumeral joint. Nevertheless, their conclusions were based on cross-correlation analysis of the shoulder muscle activity measured without any condition of glenohumeral instability. Consequently, to the best of our knowledge, either with cadaveric or EMG methods, no study has clearly identified which muscles enable to stabilize glenohumeral joint during a lifting task. An analysis based on quantification of shoulder muscle forces with and without glenohumeral stability during a lifting task may provide relevant information on the muscles directly involved in glenohumeral stability.

Simulations based on musculoskeletal models may be an alternative approach, since some models include glenohumeral stability constraint, namely, the resultant of the reaction force between the humeral head and the scapula should point toward the glenoid cavity [20–22]. Dickerson et al. [23] used thresholds of glenohumeral dislocation in eight directions estimated from the ratio between the shear and compressive reaction forces between the glenoid fossa and the humeral head to ensure glenohumeral stability. Using such models, Steenbrink et al. [6] assessed the function of shoulder muscles in glenohumeral stability with different rotator cuff tears during static arm elevations, nevertheless, no information is given for healthy people.

Therefore, this study aimed to assess the stabilization function of the shoulder muscles in a healthy population during a lifting task. This repetitive task, usually performed in manufactory industry, may result in glenohumeral instability because of the main activation of the anterior and middle deltoids [9, 10]. We hypothesized that the glenohumeral stability constraint led to a greater force produced by the rotator cuff muscles and the latissimus dorsi in comparison to the condition without stability constraint.

## Materials and methods

The study was approved by the “Comité d’éthique de la recherche en santé (CERES)” (Montréal, QC, Canada) (N°11-068-CERSS-D). All the participants provided a written informed consent before the experimentation.

### Participants

Thirteen healthy participants (nine male: [mean ± SD] age, 25.8 ± 1.61 years; height, 1.81 ± 0.08 m; mass, 74.4 ± 11.9 kg; arm length, 35.1 ± 2.4 cm; forearm length, 27.0 ± 1.8 cm and four female: age, 21.8 ± 1.26 years; height, 1.68 ± 0.02 m; mass, 57.3 ± 2.2 kg; arm length, 33.9 ± 1.5 cm; forearm length, 24.5 ± 1.5 cm) right-handed subjects volunteered in this study. None of the participants presented current or previous shoulder, elbow or wrist injuries.

### Instrumentation and data collection

**Fig 1:**
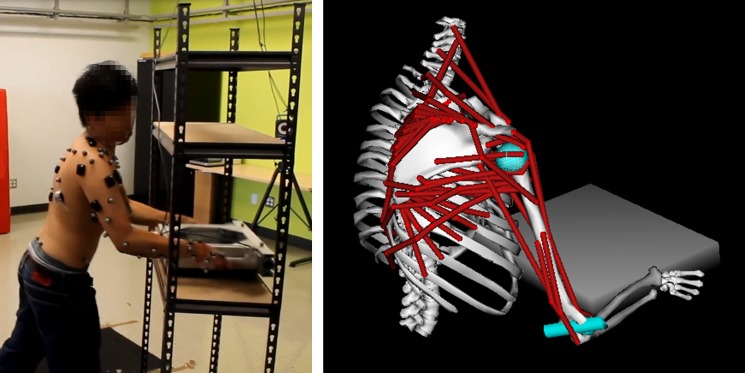
Picture of the lifting task performed by a representative participant (left) and representation of the OpenSim musculoskeletal model of the upper limb. Twenty-five reflective skin markers were placed on the thorax (xiphoid process, 3 markers on the manubrium, 1st and 10ththoracic vertebrae), on the right clavicle (sterno-clavicular joint, acromio-clavicular joint), scapula (acromion tip, acromial angle, inferior angle, trigonum spinae, superior angle), humerus (lateral and medial epicondyles), forearm (ulnar and radial styloid process), hand (proximal part of the 2nd and 3rd metacarpus, distal part of the 2nd and 5th metacarpus), and on the box used for the lifting task (four superior angles).

Only the right (dominant) side of each participant was evaluated during the box lifting task, assuming that the right and left sides of the upper body moved symmetrically [24]. Based on the upper-limb kinematic model developed by Jackson et al. [25], 25 reflective skin markers were placed on the thorax, right clavicle, scapula, humerus, forearm, hand, and on the box used for the lifting task (Fig 1). Trials were recorded using an 18-camera Vicon^TM^ motion analysis system at 200 Hz (Oxford Metrics Ltd, Oxford, UK). Surface EMG electrodes (Trigno^TM^ EMG Wireless System, Delsys, USA; 1000 Hz) were positioned on nine muscles: biceps and triceps brachialis; anterior; middle and posterior deltoid; latissimus dorsi superior head; upper, middle and lower trapezius, according to SENIAM recommendations [26].

The experimental test consisted of lifting a box (39.5 x 34.5 x 8 cm; 6 kg) positioned on a shelf from hip level to a shelf located at shoulder level. To standardize the grip of the box and ensure symmetrical movement, cylindrical handles (Ø 45 mm) were horizontally positioned on each side of the box. A 3D handle force sensor (SH2653-1106B3, Sensix, Poitiers, France; 2000 Hz) measured the 3D forces and torques between the box and the right hand. Kinematic, kinetic and EMG signals were synchronized using Nexus 1.8.2 software (Vicon, Oxford, UK).

### Experimental procedures

To normalize the experimental EMG signals recorded during the lifting and to compare the latter to the musculoskeletal activations tasks (see below), two trials of 5 s isometric maximal voluntary contraction with verbal encouragement for each of the nine muscles were performed in random order by the participants in line with the recommendations of Dal Maso et al. [27]. The participants had to exert maximum force against an experimenter during five seconds with verbal encouragement. Two trials per muscle were performed and the rest interval was 30 seconds between repetitions and 60 seconds between trials for different muscles.

To get familiarized with the box lifting procedure, participants performed a total of 10 practice sagittal lifting movements. Finally, three trials were recorded with 30 s rest in-between, without any instruction about the speed and the handgrip technique. The participants chose their preferred horizontal distance from the shelves and were instructed to limit their foot displacements to one step back.

### Data processing

After having analyzed the frequency and residual of the raw signals [28], EMG raw signal was band-pass filtered using a recursive 2^nd^-order Butterworth band-pass filter (15–500 Hz) [29]. The root mean square (RMS) EMG was calculated on a 250-ms sliding window [30]. The RMS EMG of each muscle calculated for the lifting task was normalized in amplitude with respect to the maximum RMS EMG amplitude obtained from the maximal voluntary contractions.

### Musculoskeletal model

We used an OpenSim generic model of the shoulder adapted to lifting task investigation (fully described in [9]) which was based on a combination of previous Opensim models [31–33]. Briefly the model was composed of 9 segments and 22 degrees-of-freedom and actuated by 22 muscle-tendon units (including 47 lines of actions with the muscle properties described in [34]) and 22 residual torque actuators (i.e. additional torque performed by the joint when the muscles cannot produce the joint torque required) (Fig 1). Assuming, the bilateral symmetry of the lifting movement [24], only the right upper-limb was modelled. The generic model was scaled to match each subject’s anthropometry using the experimental positions of the skin markers during a static pose. In addition, muscle maximal isometric forces were optimized for each subject-scaled-model by minimizing the difference between the normalized EMG and simulated muscle activations obtained during the lifting movement according to the method developed by Blache et al. [9].

### Stability and instability simulations

Joint angles were obtained using an inverse kinematics procedure with a point-to-ellipsoid constraint to enforce the scapula to follow the thoracic curvature throughout the movement [35, 36]. The ellipsoid was locally fitted [35, 36] and only the technical skin markers defined in Jackson et al. [25] were considered. In addition, ranges of motion were controlled by implementing boundaries for each degree of freedom obtained from data free from soft-tissue artefact [37]. These joint kinematics and the handle forces/moments were introduced into two static optimization algorithms to estimate the muscle activations and forces required to perform the lifting task in the glenohumeral instability and stability conditions.

The first algorithm was performed from the graphical user interface of OpenSim 3.2 [38]. At each time sample, the quadratic sum of muscle activations and residuals torques were minimized, such that the sum of the muscle moments is equal to the torques computed from the inverse dynamic procedure. In the second algorithm an inequality constraint was added to ensure glenohumeral stability using dislocation thresholds (ratio of shear and compression glenohumeral joint forces) determined in eight directions on cadavers [23, 39]. The joint reaction force was calculated using the *jointReaction* algorithm of OpenSim 3.2 [38, 40] and the glenohumeral dislocation thresholds of [23] were expressed as eight inequality linear constraints using a polygon of friction [41]. To summarize, in the first algorithm, lifting movements were simulated with an instability of the glenohumeral joint, while in the second algorithm, the movements were simulated with a constraint ensuring the stability of the glenohumeral joint.

### Evaluation of the model outputs with the stability constraint

Considering that the model with the glenohumeral stability constraint was the closest to human body mechanics, the outputs of the model were assessed by computing the RMS differences between normalized RMS EMG and the modelled muscle activations. Since some modelled muscles have up to five fascicles (biceps and triceps brachialis; upper, middle and lower trapezius), their mean activations were compared to the corresponding normalized EMG. Among the three trials performed by the participants, the trial with the lowest RMS difference was kept for further analysis. Finally, residual torques, which represent the capacity of the model to produce joint torques solely with its muscles were reported.

### Analysis

The ratio between the duration for which the glenohumeral reaction force was outside the friction polygon and the total trial duration was calculated for the two conditions (with and without stability constraint). The mean muscle activations and forces resulting from the static optimization with and without the stability constraint were reported.

### Statistics

The effect of the glenohumeral stability constraint on the mean residual torques; shear and compressive forces; ratio between the shear and compressive forces; muscle activations and forces was tested using linear mixed models. Linear mixed-model is an alternative method to the ANOVA on repeated measures that may be more advantageous especially with categorical data (Barr Levy, Scheepers, & Tily, 2013). As within-participants repeated measures were performed, participants were entered as random intercept. The p-values were obtained by likelihood ratio tests of full model (H_1_) against the model without the effect of the glenohumeral stability constraint (H_0_). The level of significance was initially set a p<0.05. Nevertheless, as 22 muscles were tested, a Bonferroni correction was applied (only for muscle activations and forces) to the initial level of significance that was finally set at p<0.002 (0.05/22). The linearity, homoscedasticity and normality of the residuals were graphically controlled. All analyses were executed using R software (R 3.2, RCore Team 2014, package *lme4*).

## Results

### Evaluation of the model outputs with the stability constraint

Residual mean torques were equal to 1.13 ± 0.80 N·m on average. Particularly, the residual mean torques for the three glenohumeral rotations (plane of elevation, elevation and rotation) were equal 0.22 ± 0.29 N·m, 0.15 ± 0.21 N·m and 0.41 ± 0.42 N·m, respectively (S1 Fig). The average RMS difference between the normalized EMG and the muscle model activations was 12.6 ± 6.3% of the maximal activation. (S2 Fig). The constraint of stability was respected throughout the simulated movement.

### Glenohumeral stability constraint

**Fig 2:**
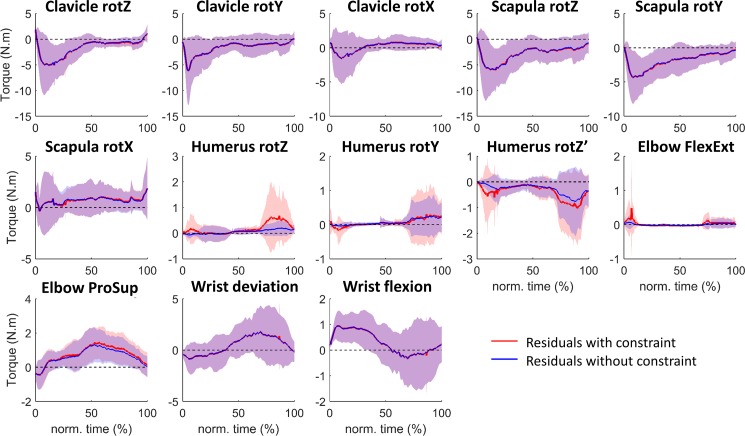
Average (±1 standard deviation represented by the shaded envelop) residual torques obtained with (in red) and without (in blue) glenohumeral stability constraint for all the participants (n = 13). Time is normalized with respect to trial duration.

All individual data are presented in supplementary materials (S1 File). The residual torques curves were similar with and without glenohumeral stability constraint (Fig 2). Furthermore, significant difference in the mean residual torques (with vs. without glenohumeral stability constraints) were only found for axial rotation of the humerus and elbow prono-supination (p = 0001; difference = 0.11 ± 0.10 Nm) and could be considered as negligible.

**Fig 3:**
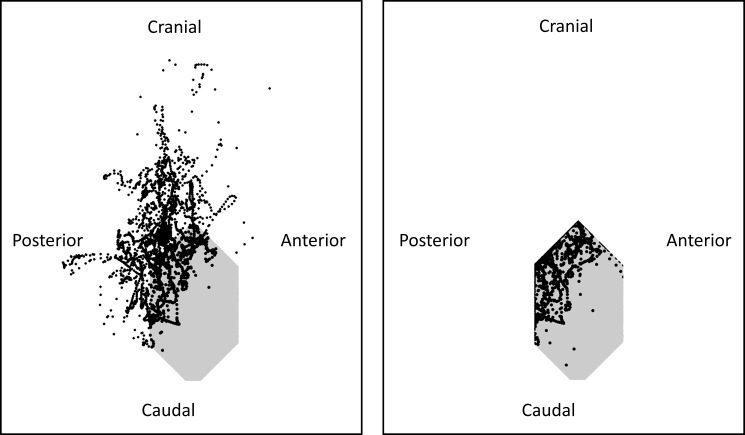
Representation of the friction polygon (in grey) and the ratio between the shear (postero-anterior and caudal-cranial) and compression forces (black cross) with (left) and without (right) glenohumeral stability constraint from all the participants (n = 13). The stability of the glenohumeral joint was ensured when the ratio was inside the friction polygon.

In the case without stability constraint, 74.0 ± 16.0% of the total lifting duration corresponded to glenohumeral reaction forces outside of the friction polygon, especially in an upward and backward direction (Fig 3).

**Fig 4:**
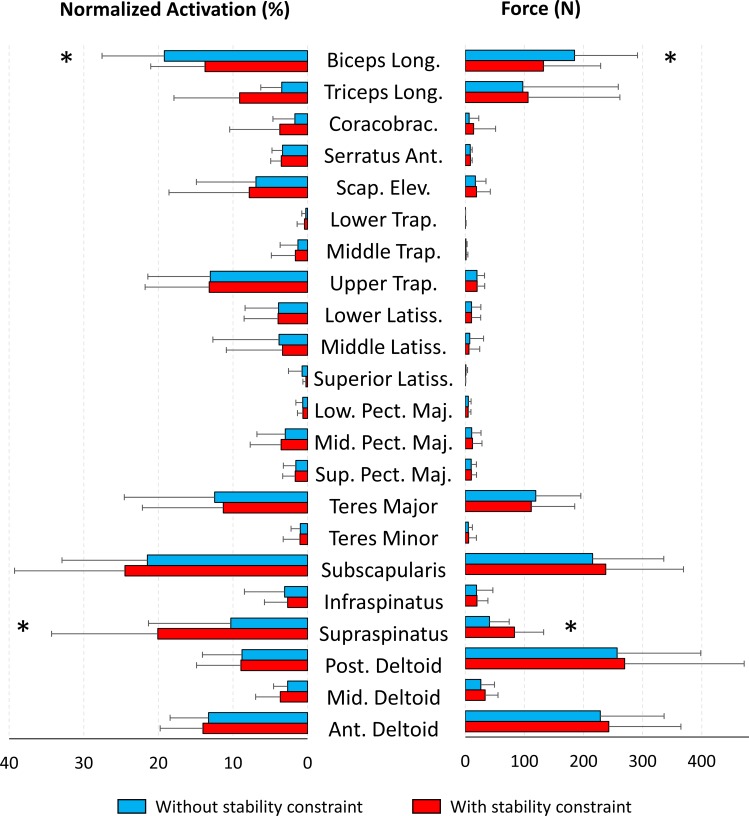
Mean normalized activations (left) and muscle forces (right) obtained with the musculoskeletal model with (in red) and without (in blue) stability constraint from all the participants (n = 13). Notes: Coracobrac: Coracobrachialis; Scap. Elev.: Scapular Elevator; Trap.: Trapezius; Latiss: Latissimus; Pect: Pectoralis; Sup: Superior; Mid: Middle; Low: Lower, Post: Posterior; Mid: Middle; Ant: Anterior. * means that the difference was significative with a p-value < 0.002.

The implementation of the glenohumeral stability constraint resulted in a significant greater mean normalized activation of the supraspinatus compared to the model without glenohumeral stability constraint (10 ± 11% vs. 20 ± 14%; χ^2^(4) = 16.4, p<0.002, power = 0.99), while the normalized activity of the biceps long head was significantly decreased (19 ± 8% vs. 14 ± 3%; χ^2^(4) = 10.2, p<0.002, power = 0.93) (Fig 4). The other mean muscle activations remained similar with *vs*. without glenohumeral stability constraint, but tendencies were found for the serratus anterior (p = 0.01, power = 0.77) and the triceps brachialis long head (p = 0.004, power = 0.90). The change in muscle activations resulted in a mean force of the supraspinatus on average two times larger due to the glenohumeral stability constraint (41 ± 34 N vs. 83 ± 49 N, χ^2^(4) = 15.0, p<0.002, power = 0.99), while the mean force was about 50 N lower for the biceps long head (185 ± 107 N vs. 132 ± 97 N, χ^2^(4) = 10.9, p<0.002, power = 0.95) (Fig 4). The other muscle mean forces remained similar with and without glenohumeral stability constraint, but tendencies were found for the pectoralis major (p = 0.008, power = 0.77), middle trapezius (p = 0.004, power = 0.86), serratus anterior (p = 0.01, power = 0.72) and triceps brachialis long head (p = 0.004, power = 0.90).

**Fig 5:**
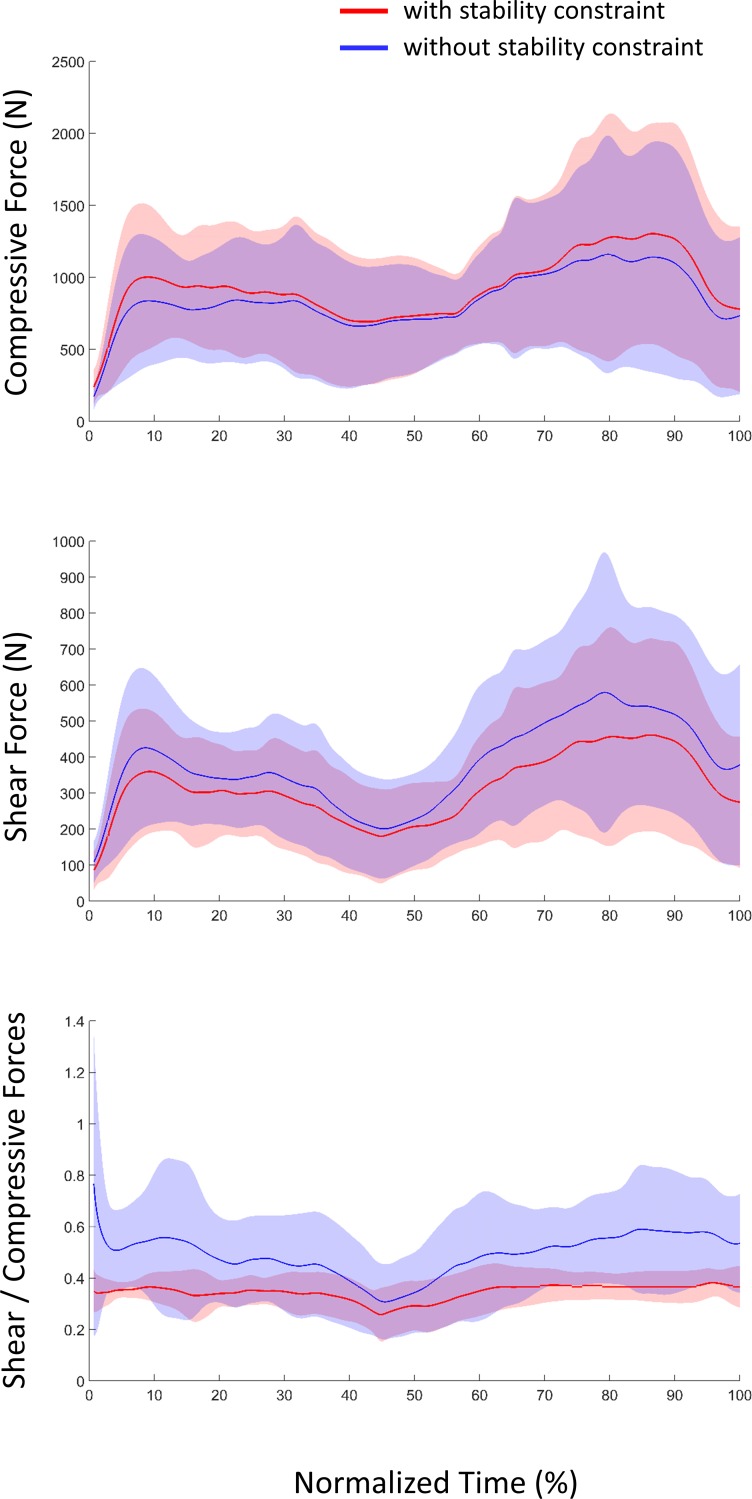
Average (±1 standard deviation represented by the shaded envelop) glenohumeral force oriented toward and orthogonally to the glenoid fossa (compressive force in newtons–Top) and tangential to the glenoid fossa, namely, in anterior-posterior and cranio-caudal directions (shear force in newtons—middle). Ratio between the shear and compression glenohumeral forces (bottom). Red and blue curves correspond to the condition with and without stability constraint respectively for all the participants (n = 13). Time is normalized with respect to trial duration, corresponding to the time from the onset of the lifting movement to the release of the box.

The mean compressive force was significantly greater (p<0.01, power = 0.90) with the implementation of the glenohumeral stability constraint and the shear forces significantly lower (p<0.001, power = 0.99). Consequently, the mean ratio between shear and compressive forces was significantly reduced with glenohumeral stability constraint (p<0.001, power = 0.99) (Fig 5).

## Discussion

Our objective was to assess stabilization function of shoulder muscles during a lifting task using an upper-limb musculoskeletal model with *vs*. without glenohumeral stability constraint. Our hypothesis was partially verified since only the supraspinatus among rotator cuff muscles was sensitive to the glenohumeral stability constraint. We may assume that the supraspinatus has a significant function in the glenohumeral stabilization during lifting task.

### Evaluation of the model outputs with the stability constraint

Using static optimization, musculoskeletal model outputs depend mainly on the measured marker trajectories [42]. Although we cannot quantify the accuracy of the shoulder kinematics in the present study, we have used state-of-the-art methods that enable the limitation of the negative effects of tissue artifact. Indeed, multibody kinematics optimization with Jackson et al. [25] technical markers combined to an ellipsoid constraint should lead to error inferior to 10° and 5° for the scapulo-thoracic and humerothoracic joints respectively [42].

Although the musculoskeletal model was not subject-specific but scaled, it respected the salient characteristics of the participants’ lifting movements. Firstly, the residual torques were low compared to the torques computed from inverse dynamics (e.g. residuals < 0.41 N·m for the glenohumeral joint), meaning that the joint torques were mainly ensured by the modelled muscles. However, elbow pro-supination and wrist joint torques were produced by the residual torque actuators due to the absence of modeled muscles implemented around the elbow and wrist joints. Since the upper-limb is a kinetic chain, the absence of wrist and some elbow modeled muscles may lead to discrepancies between modeled and *in-vivo* muscle forces. Nevertheless, all bi-articular muscles, such as the biceps brachii, crossing both the glenohumeral joint and the wrist have been implemented in the model. Consequently, we believe that all muscles crossing the glenohumeral joint play their part into the shoulder/upper-limb chain, limiting therefore the negative effect of virtual torque actuators on shoulder muscle estimate.

Secondly, similarly to Hawkes et al. [19], we observed that among the superficial muscles, the musculoskeletal model yielded greater averaged activations of the upper trapezius and anterior deltoid in comparison to the posterior deltoid and latissimus dorsi. Nevertheless, even if the modeled activations and EMG amplitude were close (12% of difference on average), the musculoskeletal model underestimated muscle activations as in Huang et al. [43] because of the least activation criterion. The co-activation between agonist and antagonist muscles is limited to the glenohumeral stability condition. For example, the activations of the trapezius and serratus anterior that ensure the scapula stabilization using co-contraction [44] were underestimated (S2 Fig). Although assessing the effect of such an underestimation on our outcomes is not straightforward, it may be limited since trapezius and serratus anterior are not inserted on the humerus and do not affect directly glenohumeral joint stability. Advanced musculoskeletal algorithms using the EMG as input [45] could be implemented in future studies.

Finally, we did not compare the simulated glenohumeral joint reaction forces to experimental data. Nevertheless, a previous study [46] based on the Delft Shoulder and Elbow model put forward that the glenohumeral joint reaction forces computed with their model were similar to those measured in patients for similar shoulder ranges of motion [47]. The simulated glenohumeral joint reaction forces measured in our study were slightly greater than those observed in their patient group but in the same order of magnitude (933±205 N vs. ~650 N, respectively). These discrepancies may be due to the higher load lifted in our study (6 kg vs. 0 kg). Consequently, we may assume that our glenohumeral reaction forces were realistic.

### Modeled glenohumeral stability and instability

From a clinical point of view, glenohumeral joint instability has been defined as a too large displacement that may lead to joint dislocation in an extreme case [3, 5]. Therefore, using a musculoskeletal model with the implementation of a constraint of glenohumeral stability based on dislocation thresholds seems to be relevant to simulate glenohumeral stability or instability. Threshold values of Dickerson et al. [23] implemented as inequality linear constraints as proposed in Wieber [48] enabled to respect glenohumeral stability with an efficient computing approach. Indeed, 100% of the glenohumeral joint reaction forces were inside the polygon friction when the constraint was implemented compared to about 25% without the constraint.

A limitation related to our musculoskeletal model was that glenohumeral joint reaction force was only the resultant of muscle forces. Therefore, glenohumeral joint stability was only ensured by the muscles, while passive structures such as the labrum and glenohumeral ligaments may also participate to glenohumeral joint stability [12]. This simplification could lead to an overestimation of the role of the shoulder muscles in glenohumeral joint stability. However, Engin and Chen [7] observed that passive contribution of the glenohumeral ligament were negligible during the mid-range of motion and increased only from the last 20° of the humerus elevation. In our studied lifting task, the glenohumeral joint was not close to its maximal elevation, since the box was lifted from the hip to the shoulder level. Consequently, we may assume that the glenohumeral ligament had a negligible effect on glenohumeral stability. In addition, Halder et al. [2] observed that the labrum contributed to less than 10% of the glenohumeral stability whatever the direction of the humerus movement. Therefore, during the lifting task investigated here, we may assume that the contribution of the passive structures to the glenohumeral stability was small and the shoulder muscles mainly ensured that glenohumeral joint stability.

### Stabilisation function of the shoulder muscles

Only the supraspinatus developed significantly more force when the glenohumeral stability constraint was implemented. Other muscles such as the pectoralis major, middle trapezius, serratus anterior and triceps long head tended to produce more force with the glenohumeral stability constraint. Although the supraspinatus is most sensitive to the glenohumeral stability constraint, the tendency of the other muscles to produce more force denotes that the stability of the shoulder is a complex system. Indeed, the stabilization function of a given muscle is possible only with the co-activation of the other shoulder muscles [12, 49].

According to the mechanical definition of the glenohumeral stability or instability given in our introduction [3] and previous study using the same method [6], we may assume that the supraspinatus ensures a great part of the glenohumeral stability during a lifting task. This outcome is in accordance with several studies [12, 15–17, 39, 50] that pointed out that the main function of the supraspinatus is to stabilize the glenohumeral joint. The increase in the supraspinatus force would enable to decrease the glenohumeral shear forces and to increase the compressive forces (Fig 5). In addition, previous studies assumed that the direction of the supraspinatus line of action enables the latter to compress the glenohumeral head against the glenoid fossa [3, 51], especially to act against humeral head translations caused by the deltoids muscle activations [8]. During lifting task, the anterior and middle deltoids are activated to ensure arm flexion and abduction (similar activation with and without stability constraint). Therefore, we may assume that the extra activation of the supraspinatus is necessary to overcome the pulling effect of the deltoids, since the deltoid muscles may pull the humeral head outside the glenoid fossa.

Contrary to previous studies and our hypothesis, we did not observe that the other rotator cuff muscles and the latissimus dorsi produced more force with the stability glenohumeral constraint. One explanation may be that during the lifting task most of the glenohumeral forces pointed in a backward and upward directions (Fig 3), consequently the stabilization function of the subscapularis (i.e. to limit anteroposterior translations of the humeral head [12]) may be limited. The force produced by the subscapularis with and without the stability constraint was probably used to ensure the internal rotation of the humerus observed during the lifting phase. These results reinforce the hypothesis that the function of the rotator cuff muscles may differ depending on the direction of the arm movement [50]. Our results should however be taken with caution, because rotator cuff activations were not measured using invasive intramuscular EMG as in [52, 53].

A last limitation was that only one box dimension was chosen regardless the participant’s anthropometry and strength. In consequence, the relative load and dimension differed between participants increasing the inter-subject joint kinematics and kinetics variability. However, in manufactory industry, the mass of the box that need to be lifted are not dependent of the employee anthropometry. In consequence, only one box dimension corresponding to the manufacturing standards was used in this study such as previous investigations in ergonomics field [19, 31, 54]. Further studies are needed to assess the effect of box mass and dimension on glenohumeral stability during lifting tasks.

## Conclusion

To the best of our knowledge, this study is the first that evaluated the contribution of shoulder muscles in glenohumeral stability during dynamic movement such as a lifting task. Considering that the musculoskeletal model has reproduced the salient characteristics of the participants’ movements, the simulations with and without glenohumeral stability constraint enabled to better understand the role of stabilizing muscles during a lifting task. We observed that only the supraspinatus muscle was sensitive to the glenohumeral stability constraint. This outcome provides further evidences that the supraspinatus may be a main stabilizer of the glenohumeral joint in a healthy population during lifting task. Thus, it may be assumed that the integrity of the supraspinatus is necessary to prevent glenohumeral instability and therefore limit the risk of shoulder injuries during lifting tasks.

## Supporting information

S1 Fig Average (±1 standard deviation represented by the shaded envelop) joints torques obtained with inverse dynamics (red), and residuals torques obtained with static optimization (blue) for the 13 degrees of freedom actuated by the muscles model. Time is normalized with respect to trial duration.
(DOCX)

S2 Fig Average (±1 standard deviation represented by the shaded envelop) muscle model activations obtained with static optimization (in red) and normalized EMG (in blue). Time is normalized with respect to trial duration.
(DOCX)

S1 File Individual data used to perform the statistics. (XLSX)

## References

[1] Wilk KE, Arrigo CA, Andrews JR. Current concepts: the stabilizing structures of the glenohumeral joint. J Orthop Sports Phys Ther. 1997;25(6):364–79. doi: 10.2519/jospt.1997.25.6.364 9168344 10.2519/jospt.1997.25.6.364

[2] Halder AM, Kuhl SG, Zobitz ME, Larson D, An KN. Effects of the glenoid labrum and glenohumeral abduction on stability of the shoulder joint through concavity-compression: an in vitro study. J Bone Joint Surg Am. 2001;83-A(7):1062–9. 11451977 10.2106/00004623-200107000-00013

[3] Veeger HE, van der Helm FC. Shoulder function: the perfect compromise between mobility and stability. J Biomech. 2007;40(10):2119–29. doi: 10.1016/j.jbiomech.2006.10.016 17222853 10.1016/j.jbiomech.2006.10.016

[4] Pagnani MJ, Warren RF. Stabilizers of the glenohumeral joint. J Shoulder Elbow Surg. 1994;3(3):173–90. doi: 10.1016/S1058-2746(09)80098-0 22959695 10.1016/S1058-2746(09)80098-0

[5] Soslowsky LJ, Malicky DM, Blasier RB. Active and passive factors in inferior glenohumeral stabilization: a biomechanical model. J Shoulder Elbow Surg. 1997;6(4):371–9. 9285877 10.1016/s1058-2746(97)90005-7

[6] Steenbrink F, de Groot JH, Veeger HE, van der Helm FC, Rozing PM. Glenohumeral stability in simulated rotator cuff tears. J Biomech. 2009;42(11):1740–5. doi: 10.1016/j.jbiomech.2009.04.011 19450803 10.1016/j.jbiomech.2009.04.011

[7] Engin AE, Chen SM. Statistical data base for the biomechanical properties of the human shoulder complex—II: Passive resistive properties beyond the shoulder complex sinus. J Biomech Eng. 1986;108(3):222–7. 3747466 10.1115/1.3138606

[8] Escamilla RF, Yamashiro K, Paulos L, Andrews JR. Shoulder muscle activity and function in common shoulder rehabilitation exercises. Sports Med. 2009;39(8):663–85. doi: 10.2165/00007256-200939080-00004 19769415 10.2165/00007256-200939080-00004

[9] Blache Y, Desmoulins L, Allard P, Plamondon A, Begon M. Effects of height and load weight on shoulder muscle work during overhead lifting task. Ergonomics. 2015;58(5):748–61. doi: 10.1080/00140139.2014.980336 25403553 10.1080/00140139.2014.980336

[10] Yoon J, Shiekhzadeh A, Nordin M. The effect of load weight vs. pace on muscle recruitment during lifting. Appl Ergon. 2012;43(6):1044–50. doi: 10.1016/j.apergo.2012.03.004 22475433 10.1016/j.apergo.2012.03.004

[11] Blasier RB, Soslowsky LJ, Malicky DM, Palmer ML. Posterior glenohumeral subluxation: active and passive stabilization in a biomechanical model. J Bone Joint Surg Am. 1997;79(3):433–40. 9070535

[12] Hess SA. Functional stability of the glenohumeral joint. Man Ther. 2000;5(2):63–71. doi: 10.1054/math.2000.0241 10903581 10.1054/math.2000.0241

[13] Steenbrink F, de Groot JH, Veeger HE, Meskers CG, van de Sande MA, Rozing PM. Pathological muscle activation patterns in patients with massive rotator cuff tears, with and without subacromial anaesthetics. Man Ther. 2006;11(3):231–7. doi: 10.1016/j.math.2006.07.004 16890886 10.1016/j.math.2006.07.004

[14] Labriola JE, Lee TQ, Debski RE, McMahon PJ. Stability and instability of the glenohumeral joint: the role of shoulder muscles. J Shoulder Elbow Surg. 2005;14(1 Suppl S):32S–8S. 15726085 10.1016/j.jse.2004.09.014

[15] Ward SR, Hentzen ER, Smallwood LH, Eastlack RK, Burns KA, Fithian DC, et al Rotator cuff muscle architecture: implications for glenohumeral stability. Clin Orthop Relat Res. 2006;448:157–63. doi: 10.1097/01.blo.0000194680.94882.d3 16826111 10.1097/01.blo.0000194680.94882.d3

[16] Kelly BT, Backus SI, Warren RF, Williams RJ. Electromyographic analysis and phase definition of the overhead football throw. Am J Sports Med. 2002;30(6):837–44. doi: 10.1177/03635465020300061401 12435650 10.1177/03635465020300061401

[17] Kronberg M, Nemeth G, Brostrom LA. Muscle activity and coordination in the normal shoulder. An electromyographic study. Clin Orthop Relat Res. 1990;(257):76–85. 2379377

[18] Alpert SW, Pink MM, Jobe FW, McMahon PJ, Mathiyakom W. Electromyographic analysis of deltoid and rotator cuff function under varying loads and speeds. J Shoulder Elbow Surg. 2000;9(1):47–58. 10717862 10.1016/s1058-2746(00)90009-0

[19] Hawkes DH, Alizadehkhaiyat O, Fisher AC, Kemp GJ, Roebuck MM, Frostick SP. Normal shoulder muscular activation and co-ordination during a shoulder elevation task based on activities of daily living: an electromyographic study. J Orthop Res. 2012;30(1):53–60. doi: 10.1002/jor.21482 21674607 10.1002/jor.21482

[20] van der Helm FC. A finite element musculoskeletal model of the shoulder mechanism. J Biomech. 1994;27(5):551–69. 8027090 10.1016/0021-9290(94)90065-5

[21] Blana D, Hincapie JG, Chadwick EK, Kirsch RF. A musculoskeletal model of the upper extremity for use in the development of neuroprosthetic systems. J Biomech. 2008;41(8):1714–21. doi: 10.1016/j.jbiomech.2008.03.001 18420213 10.1016/j.jbiomech.2008.03.001 PMC2586642

[22] Chadwick EK, Blana D, van den Bogert AJ, Kirsch RF. A real-time, 3-D musculoskeletal model for dynamic simulation of arm movements. IEEE Trans Biomed Eng. 2009;56(4):941–8. doi: 10.1109/TBME.2008.2005946 19272926 10.1109/TBME.2008.2005946 PMC2971671

[23] Dickerson CR, Chaffin DB, Hughes RE. A mathematical musculoskeletal shoulder model for proactive ergonomic analysis. Comput Methods Biomech Biomed Engin. 2007;10(6):389–400. doi: 10.1080/10255840701592727 17891574 10.1080/10255840701592727

[24] Nielsen PK, Andersen L, Jorgensen K. The muscular load on the lower back and shoulders due to lifting at different lifting heights and frequencies. Appl Ergon. 1998;29(6):445–50. 9796790 10.1016/s0003-6870(98)00005-2

[25] Jackson M, Michaud B, Tetreault P, Begon M. Improvements in measuring shoulder joint kinematics. J Biomech. 2012;45(12):2180–3. doi: 10.1016/j.jbiomech.2012.05.042 22748323 10.1016/j.jbiomech.2012.05.042

[26] Hermens HJ, Freriks B, Disselhorst-Klug C, Rau G. Development of recommendations for SEMG sensors and sensor placement procedures. J Electromyogr Kinesiol. 2000;10(5):361–74. 11018445 10.1016/s1050-6411(00)00027-4

[27] Dal Maso F, Marion P, Begon M. Optimal Combinations of Isometric Normalization Tests for the Production of Maximum Voluntary Activation of the Shoulder Muscles. Arch Phys Med Rehabil. 2016;97(9):1542–51 e2. doi: 10.1016/j.apmr.2015.12.024 26801059 10.1016/j.apmr.2015.12.024

[28] Winter DA. Biomechanics and Motor Control of Human Movement. Fourth ed. Hoboken, New Jersey: John Wiley and Sons; 2009.

[29] De Luca CJ, Gilmore LD, Kuznetsov M, Roy SH. Filtering the surface EMG signal: Movement artifact and baseline noise contamination. J Biomech. 2010;43(8):1573–9. doi: 10.1016/j.jbiomech.2010.01.027 20206934 10.1016/j.jbiomech.2010.01.027

[30] Merletti R. Standards for reporting EMG Data. J Electromyogr Kinesiol. 1999;9(1):3–4.

[31] Steele T, Merryweather A, Dickerson CR, Bloswick D. A computational study of shoulder muscle forces during pushing tasks. Int J Human Factors Modelling and Simulation. 2013;4(1):1–22.

[32] Holzbaur KR, Murray WM, Delp SL. A model of the upper extremity for simulating musculoskeletal surgery and analyzing neuromuscular control. Ann Biomed Eng. 2005;33(6):829–40. 16078622 10.1007/s10439-005-3320-7

[33] Nikooyan AA, Veeger HE, Chadwick EK, Praagman M, Helm FC. Development of a comprehensive musculoskeletal model of the shoulder and elbow. Med Biol Eng Comput. 2011;49(12):1425–35. doi: 10.1007/s11517-011-0839-7 22038240 10.1007/s11517-011-0839-7 PMC3223593

[34] Thelen DG. Adjustment of muscle mechanics model parameters to simulate dynamic contractions in older adults. J Biomech Eng. 2003;125(1):70–7. 12661198 10.1115/1.1531112

[35] Michaud B, Begon B. Thorax ellipsoid optimization based on scapula movements' area improves kinematic reconstruction of the scapula. Digital Human Modeling Congress; Montreal Canada2016, July. p. 26.

[36] Seth A, Matias R, Veloso AP, Delp SL. A Biomechanical Model of the Scapulothoracic Joint to Accurately Capture Scapular Kinematics during Shoulder Movements. PLoS One. 2016;11(1):e0141028 doi: 10.1371/journal.pone.0141028 26734761 10.1371/journal.pone.0141028 PMC4712143

[37] Ludewig PM, Phadke V, Braman JP, Hassett DR, Cieminski CJ, LaPrade RF. Motion of the shoulder complex during multiplanar humeral elevation. J Bone Joint Surg Am. 2009;91(2):378–89. doi: 10.2106/JBJS.G.01483 19181982 10.2106/JBJS.G.01483 PMC2657311

[38] Delp SL, Anderson FC, Arnold AS, Loan P, Habib A, John CT, et al OpenSim: open-source software to create and analyze dynamic simulations of movement. IEEE Trans Biomed Eng. 2007;54(11):1940–50. doi: 10.1109/TBME.2007.901024 18018689 10.1109/TBME.2007.901024

[39] Lippitt S, Matsen F. Mechanisms of glenohumeral joint stability. Clin Orthop Relat Res. 1993;(291):20–8. 8504601

[40] Steele KM, Demers MS, Schwartz MH, Delp SL. Compressive tibiofemoral force during crouch gait. Gait Posture. 2012;35(4):556–60. doi: 10.1016/j.gaitpost.2011.11.023 22206783 10.1016/j.gaitpost.2011.11.023 PMC3319529

[41] Blache Y, Creveaux T, Dumas R, Cheze L, Rogowski I. Glenohumeral contact force during flat and topspin tennis forehand drives. Sports Biomech. 2017;16(1):127–42. doi: 10.1080/14763141.2016.1216585 27595163 10.1080/14763141.2016.1216585

[42] Blache Y, Begon B. Influence of shoulder kinematic estimate on joint and muscle mechanics predicted by musculoskeletal model. IEEE Trans Biomed Eng. In Press. 10.1109/TBME.2017.2716186 28641241

[43] Huang L, Zhuang J, Zhang Y. The application of computer musculoskeletal modeling and simulation to investigate compressive tibiofemoral force and muscle functions in obese children. Comput Math Methods Med. 2013;2013:305434 doi: 10.1155/2013/305434 24288573 10.1155/2013/305434 PMC3833069

[44] Noguchi M, Chopp JN, Borgs SP, Dickerson CR. Scapular orientation following repetitive prone rowing: implications for potential subacromial impingement mechanisms. J Electromyogr Kinesiol. 2013;23(6):1356–61. doi: 10.1016/j.jelekin.2013.08.007 24055533 10.1016/j.jelekin.2013.08.007

[45] Belaise C, Dal Maso F, Michaud B, Mombaur K, Begon M. An EMG-marker tracking optimisation for estimating muscle forces. Multibody System Dynamics. 2017 doi: 10.1007/s11044-016-9541-8

[46] Veeger HE, Yu B, An KN, Rozendal RH. Parameters for modeling the upper extremity. J Biomech. 1997;30(6):647–52. 9165401 10.1016/s0021-9290(97)00011-0

[47] Nikooyan AA, Veeger HE, Westerhoff P, Graichen F, Bergmann G, van der Helm FC. Validation of the Delft Shoulder and Elbow Model using in-vivo glenohumeral joint contact forces. J Biomech. 2010;43(15):3007–14. doi: 10.1016/j.jbiomech.2010.06.015 20655049 10.1016/j.jbiomech.2010.06.015

[48] Wieber PB, editor On the stability of walking systems. International Workshop on Humanoid and Human Friendly Robotics; 2002; Tsukuba, Japan

[49] Blache Y, Dal Maso F, Desmoulins L, Plamondon A, Begon M. Superficial shoulder muscle co-activations during lifting tasks: Influence of lifting height, weight and phase. J Electromyogr Kinesiol. 2015;25(2):355–62. doi: 10.1016/j.jelekin.2014.11.004 25483204 10.1016/j.jelekin.2014.11.004

[50] Wattanaprakornkul D, Cathers I, Halaki M, Ginn KA. The rotator cuff muscles have a direction specific recruitment pattern during shoulder flexion and extension exercises. J Sci Med Sport. 2011;14(5):376–82. doi: 10.1016/j.jsams.2011.01.001 21333595 10.1016/j.jsams.2011.01.001

[51] Sangwan S, Green RA, Taylor NF. Stabilizing characteristics of rotator cuff muscles: a systematic review. Disabil Rehabil. 2015;37(12):1033–43. doi: 10.3109/09638288.2014.949357 25116629 10.3109/09638288.2014.949357

[52] Heuberer P, Kranzl A, Laky B, Anderl W, Wurnig C. Electromyographic analysis: shoulder muscle activity revisited. Arch Orthop Trauma Surg. 2015;135(4):549–63. doi: 10.1007/s00402-015-2180-3 25720847 10.1007/s00402-015-2180-3

[53] Meskers CG, de Groot JH, Arwert HJ, Rozendaal LA, Rozing PM. Reliability of force direction dependent EMG parameters of shoulder muscles for clinical measurements. Clin Biomech (Bristol, Avon). 2004;19(9):913–20. 10.1016/j.clinbiomech.2004.05.012 15475123

[54] Moriguchi CS, Carnaz L, de Miranda LC Jr., Marklin RW, Coury HJ. Biomechanical analysis of loading/unloading a ladder on a truck. Work. 2012;41 Suppl 1:2492–5. 22317093 10.3233/WOR-2012-0487-2492

