# Supplementary material for: Muscle function in glenohumeral joint stability during lifting task
Source: PLoS One. 2017 Dec 15;12(12):e0189406. doi: 10.1371/journal.pone.0189406 (PMC5731701; doi:10.1371/journal.pone.0189406)

**S1 Fig** Average (±1 standard deviation represented by the shaded envelop) joints torques obtained with inverse dynamics (red), and residuals torques obtained with static optimization (blue) for the 13 degrees of freedom actuated by the muscles model. Time is normalized with respect to trial duration.


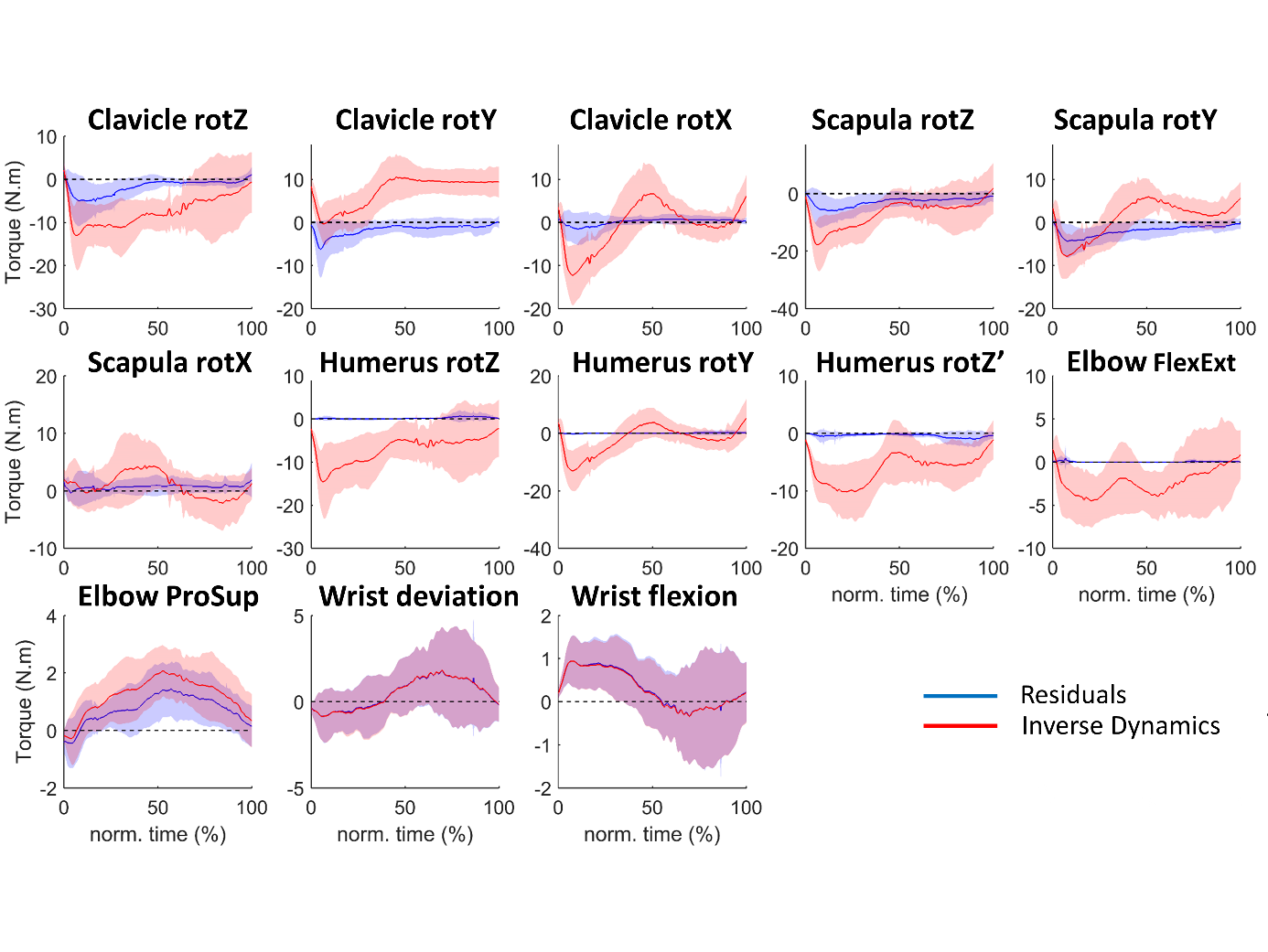

Supplement: S1 Fig — Time is normalized with respect to trial duration. (DOCX) [file pone.0189406.s001.DOCX]
