# Supplementary material for: Muscle function in glenohumeral joint stability during lifting task
Source: PLoS One. 2017 Dec 15;12(12):e0189406. doi: 10.1371/journal.pone.0189406 (PMC5731701; doi:10.1371/journal.pone.0189406)

**S2 Fig** Average (±1 standard deviation represented by the shaded envelop) muscle model activations obtained with static optimization (in red) and normalized EMG (in blue). Time is normalized with respect to trial duration.


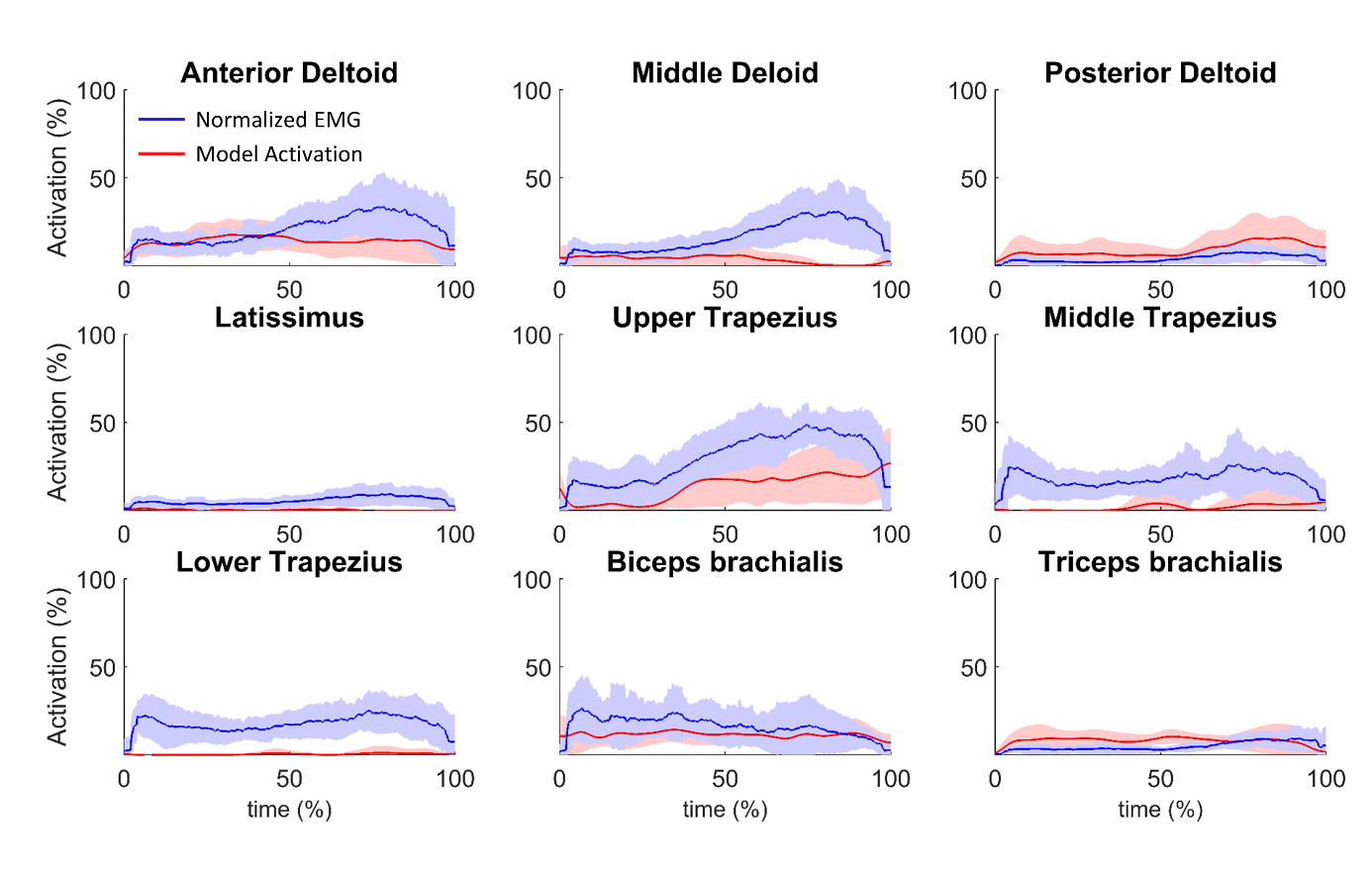

Supplement: S2 Fig — Time is normalized with respect to trial duration. (DOCX) [file pone.0189406.s002.DOCX]
